# Impact of Irradiation on Post-Surgical Residuals of WHO Grade I Meningioma

**DOI:** 10.3390/jcm14165829

**Published:** 2025-08-18

**Authors:** Alice Giotta Lucifero, Rami Almefty, Ossama Al-Mefty

**Affiliations:** 1 Department of Neurosurgery, Mass General Brigham, Harvard Medical School, Boston, MA 02115, USA; alicelucifero@gmail.com; 2 Department of Brain and Behavioral Sciences, University of Pavia, 27100 Pavia, Italy; 3 Department of Neurosurgery, Lewis Katz School of Medicine, Temple University, Philadelphia, PA 19140, USA; rami.almefty@tuhs.temple.edu

**Keywords:** brain tumor, gamma-knife, meningioma, radiotherapy, tumor malignant progression

## Abstract

**Background:** Radiotherapy is widely used to control postoperative residuals of WHO grade I meningiomas, with favorable outcomes reported from relatively short follow-ups. The aim of this study is to evaluate the effect of radiation on extended long-term outcome of benign meningiomas, comparing radiated to non-radiated post-surgical residuals. **Methods:** A retrospective observational record analysis of 2499 consecutive meningiomas treated from 1990 through 2023 identified 436 WHO grade I meningiomas with post-surgical residuals after subtotal resection (STR); of these, 176 received radiotherapy. Progression-free survival, cause-specific overall survival, and mortality were analyzed. Clinical control was defined as the absence of post-irradiation intervention. Malignant transformation was confirmed histologically. **Results:** At a median and mean follow-up of 103.5 and 127.28 months, the 3-, 5-, 10-, and 15-year progression-free survival were 91%, 85%, 77%, and 70% following STR alone, and 59%, 43%, 23%, and 16% after STR plus radiotherapy. The cause-specific overall survival at 5, 10, 15, and 20 years was 97.6%, 97.6%, 97.6%, and 96% for STR and 97%, 93%, 85%, and 76% for STR with irradiation, respectively. Mortality was 26% in the irradiated group, compared to 4%. Clinical control was achieved in 87% and 37% in the surgery and irradiation groups, respectively. Malignant transformation occurred in 28% of the irradiated group and 1% after surgery alone. **Conclusions:** This study revealed that with a follow-up beyond 10 years, irradiation of residual WHO I meningiomas was associated with increased recurrence, worse survival, less clinical control, and increased malignant progression.

## 1. Introduction

Meningiomas are the most common primary intracranial neoplasms, accounting for 39% of all brain tumors, with an incidence of 9.12 per 100,000 individuals and increasing with age [1]. The majority are benign, with 80% of newly diagnosed cases classified as WHO Grade I [1].

Surgical resection remains the most effective initial treatment, and the extent of resection is the predominant factor in reducing the recurrence rate [2,3].

Historically, benign meningiomas were considered radioresistant [4]; however, since the late 1990s, advances in irradiation modalities have enhanced the accuracy of tumor targeting, allowing for more precise dose delivery with the intent of reducing exposure to surrounding healthy tissues.

Radiation treatments became widely and commonly used to treat residual WHO grade I tumors [5,6]. Subtotal resection followed by adjuvant radiation has been associated with tumor control in several studies, albeit over a relatively short period [7].

In a recent prospective phase II trial (NRG/RTOG 0539), patients with a residual WHO grade I meningioma who did not receive postoperative irradiation had 3, 5, and 10-year progression-free survival (PFS) rates of 83.1%, 72.7%, and 72.7%, respectively, and 10-year overall survival of 100%, raising doubts about the need for irradiation in this group [8].

Our study analyzed 436 consecutive WHO grade I meningiomas in patients from a single center treated with either subtotal resection alone or with surgery and radiation. The primary aim was to assess the impact of irradiation on long-term outcomes, including tumor recurrence, clinical control, survival rates, and potential malignant transformation.

## 2. Materials and Methods

### 2.1. Study Design and Population

This retrospective observational study investigated a cohort of all record-available patients diagnosed with benign meningiomas who underwent subtotal surgical resection from January 1990 through December 2023 at the Department of Neurosurgery, Brigham and Women’s Hospital, Harvard Medical School. The study was conducted under the approval of the institutional review board [IRB: 2011P001223].

Eligibility criteria included a histopathologically confirmed WHO grade I meningioma treated with subtotal surgical resection with or without irradiation and a minimum postoperative follow-up period of 12 months. Patients diagnosed with radiation-induced meningiomas, tumors associated with genetic syndromes, or WHO grade II or III tumors were excluded from the analysis. Demographic and clinical data were systematically collected from electronic medical records. WHO grade I was applied according to the WHO criteria at the time of first surgery. For patients with multiple meningiomas, each lesion was treated as an independent entity.

### 2.2. Treatments

All patients had residual tumors after the first surgical intervention. The extent of resection was determined from the operative reports and measurements taken from quantitative imaging analysis of postoperative magnetic resonance imaging (MRI). Simpson grading could not be applied because of the lack of definition in many operative reports.

Radiation treatments were classified as fractionated stereotactic conformal radiotherapy (SFRT), stereotactic radiosurgery (SRS), and fractionated proton beam therapy (FPB). They were labeled based on timing as neoadjuvant, adjuvant, or administered at recurrence. Data regarding the number of surgical resections, radiation doses (in Gray, Gy), and postoperative clinical trials or medical protocols were recorded.

### 2.3. Follow-Up Workflow and Recurrences

Postoperative MRI was performed within 48 h of surgery, and subsequent MRIs were usually performed at 3, 6, and 12 months, then annually or biannually or when needed. Tumor recurrence was defined as radiographic progression, which included an increase in tumor volume, enhancement, or nodularity detected on post-treatment MRI scans. The late postoperative Karnofsky Performance Scale status was recorded during the last clinical visit.

### 2.4. Endpoints and Statistical Analysis

All statistical analyses were performed using R software [version 4.4.1] and JMP [version 17]. Data integrity and accuracy were ensured through rigorous checking procedures. Descriptive statistics, including the mean and standard deviation for continuous variables and frequency distributions for categorical variables, were computed for the entire cohort and stratified by treatment group. For the analysis, patients were categorized into two treatment groups: the group who underwent subtotal resection alone (STR group) and the group who received postoperative radiation therapy (STR + RT group).

The study evaluated two time-to-event primary outcomes: progression-free survival (PFS) and overall survival (OS). PFS was calculated as the time from initial surgery to the first radiological evidence of disease progression (recurrence). OS was defined as the time from the first treatment to the last follow-up or death. To account for competing risks associated with mortality from causes other than meningiomas, we performed cause-specific analysis for patients who died from meningiomas with respect to those who died from other causes. This approach ensured that survival estimates were not biased by competing mortality.

Survival rates were calculated and presented as actual median values and actuarial percentages at 3, 5, 10, 15, and 20 years. Survival curves were generated with the Kaplan–Meier method. The differences between the treatment groups were assessed with the log-rank test to determine statistical significance. A *p*-value of <0.05 was considered statistically significant.

The Kaplan–Meier method was also used to evaluate the treatment’s potential time effect, comparing the immediate versus delayed benefits of radiation therapy. Separate survival curves were constructed to compare patients who received immediate postoperative radiation therapy with those who underwent delayed or non-immediate radiotherapy.

The mortality rate was calculated across four time points (0–36, 36–60, 60–120, and 120–180 months) over 15 years of follow-up. Mortality data were retrieved from medical records and corroborated with the Social Security Death Index.

The Cox proportional hazards (Cox-PH) regression model assessed the impact of various clinical variables on OS and PFS. The covariates included tumor size and location and the patient’s age, sex, race, and treatment. The proportional hazards assumption was tested using a graphical forest plot, ensuring the model was appropriately specified. The Bonferroni multiplicity adjustment was applied to reduce error type I bias.

Clinical endpoints were defined as the common term “clinical control,” characterized by the absence of any additional therapeutic interventions after the first surgery or radiotherapy, and “malignant transformation,” determined through histopathological analysis of sequential specimen and, where available, supported by genetic studies.

## 3. Results

### 3.1. Baseline Data

The data of 2499 meningioma cases were reviewed. A total of 436 meningiomas were included in the study, since three patients had more than one tumor.

The mean patient age was 69 ± 13.9 years, with a predominance of females (73%, n. 319) and Caucasian ethnicity (83%, n. 363). All patients initially underwent subtotal resection, with an intraoperative complication rate of 2%. Of these, 176 patients (40%) received irradiation.

Table 1 provides a detailed summary of patient demographics, clinical characteristics, pathological findings, and genetic information.

### 3.2. Irradiation Protocols

**Figure 1:**
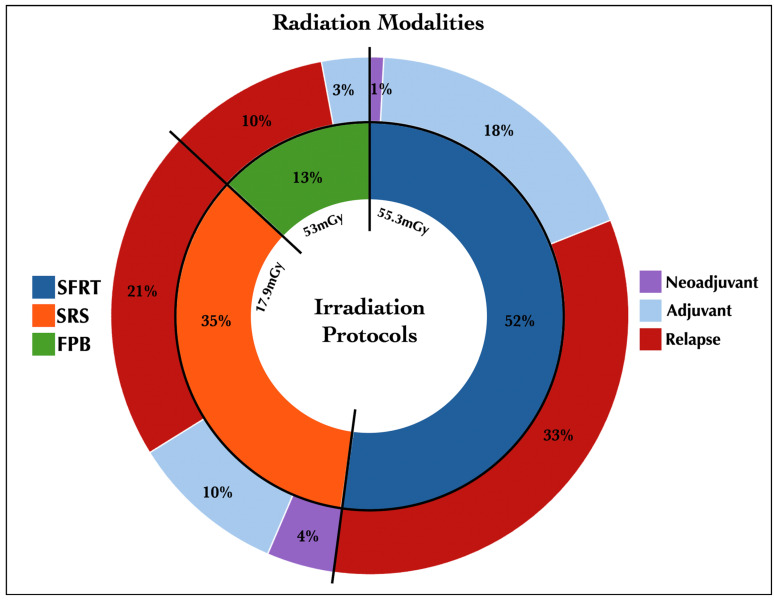
Pie chart depicting the irradiation group characters. Of the patients, 52% received SFRT, of whom 33% received it for recurrence and 18% as an adjuvant; 35% of patients received SRS, with 21% for recurrence; and 13% underwent FPB, 10% of them for recurrence. FPB, fractionated proton beam therapy; mGy, mean Gray; SFRT, fractionated stereotactic conformal radiotherapy; SRS, stereotactic radiosurgery.

A total of 237 radiation treatments were administered across 176 meningiomas, including fractionated stereotactic conformal radiotherapy in 124 cases (52%) with a mean dose of 55.3 Gy, stereotactic radiosurgery in 82 cases (35%) with a mean dose of 17.9 Gy, and fractionated proton beam therapy in 31 cases (13%) with a mean dose of 53 Gy/relative biological effectiveness (RBE) (Figure 1).

Overall, 81 patients (46%) received adjuvant radiotherapy after their initial surgical resection, while 95 (54%) underwent irradiation as a treatment for tumor recurrence. In addition, ten patients belonging to the irradiated group received adjuvant medical therapies, which included agents such as the DLB2 TEAD1-inhibitor, ASP2215 gilteritinib, DFCI 11–165 bevacizumab, DFCI 15–490 nivolumab, pembrolizumab, and SOM230C pasireotide.

### 3.3. Recurrence Rates

The median and mean clinical and radiographic follow-up durations were 103.5 and 127.28 months, respectively. In the STR group, 86 patients (33%) experienced a first recurrence, and 35 (13%) required a second intervention. In the STR + RT group, 155 patients (88%) had a first recurrence, 72 patients (41%) had a second recurrence, and 40 patients (23%) experienced a third recurrence. Among these 176 patients, 87 (49%) required a second surgery. Intervals between recurrences progressively shortened, while the mean tumor growth rate increased with each recurrence, especially for the STR + RT group (Table S1).

### 3.4. Extended Long-Term Outcomes

**Figure 2:**
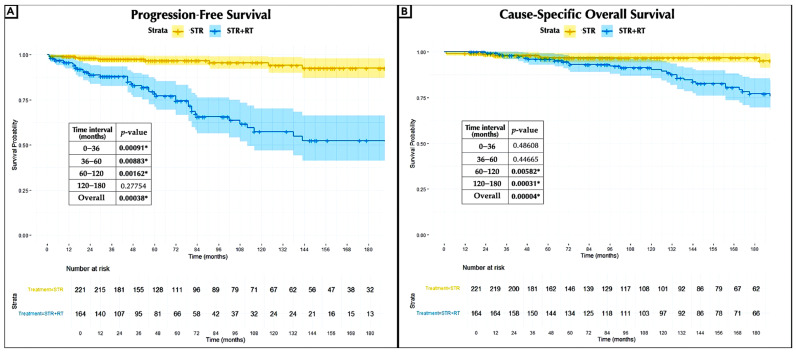
Kaplan–Meier curves of progression-free survival (**A**) and cause-specific overall survival (**B**) comparing the STR group with the STR + RT group, with the number at risk and censored. *: A *p*-value of <0.05 was considered statistically significant; RT, radiation therapy; STR, subtotal resection.

The median PFS was 59 months for the STR group and 47.5 months for the STR + RT group. The actuarial PFS rates at 3, 5, 10, and 15 years were 91%, 85%, 77%, and 70% for the STR group and 59%, 43%, 23%, and 16% for the STR + RT group, respectively. Kaplan–Meier analysis demonstrated an overall statistically significant difference in PFS between the groups (log-rank test, *p* = 0.00038), indicating a higher risk of recurrence among patients who received radiotherapy. This increased risk became statistically significant as early as 3 years of follow-up (0–36 months, *p* = 0.00091). The absence of significant differences between the groups beyond 120 months can be attributed to the reduced sample size, as all meningiomas treated with radiotherapy recurred earlier in the follow-up period (Figure 2A). The median OS was 112 months for the STR group and 150 months for the STR + RT group. In contrast, the actuarial cause-specific OS rates at 5, 10, 15, and 20 years were 98%, 97.6%, 97.6%, 97.6%, and 96% for the STR group and 98%, 97%, 93%, 85%, and 76% for the STR + RT group. The cause-specific survival analysis demonstrated a statistically significant poorer OS after irradiation (*p* = 0.00004), starting at 60 months of follow-up (60–120 months, *p* = 0.00582) (Figure 2B).

The mortality rates for the STR group at 36 months, 36–60 months, 60–120 months, and 120–180 months were 10%, 5.7%, 0%, and 0%, respectively. For the STR + RT group, the mortality rates were 21%, 18%, 16%, and 45%. The overall mortality rate in the radiation treatment group was 26% compared to 4% in the non-radiation group, proving high statistical significance (*p* = 2.52 × 10^−7^).

Malignant progression occurred in 2 patients (1%) in the surgery-only group, both progressing to WHO grade II, and in 49 patients (28%) in the STR + RT group, of whom 39 (22%) progressed from WHO grade I to WHO grade II. Progression from WHO grade I to WHO grade II to grade III, or directly to WHO grade III, occurred exclusively after radiotherapy, affecting seven patients (4%) and three patients (2%), respectively.

The extended long-term outcomes are reported in Table 2.

**Figure 3:**
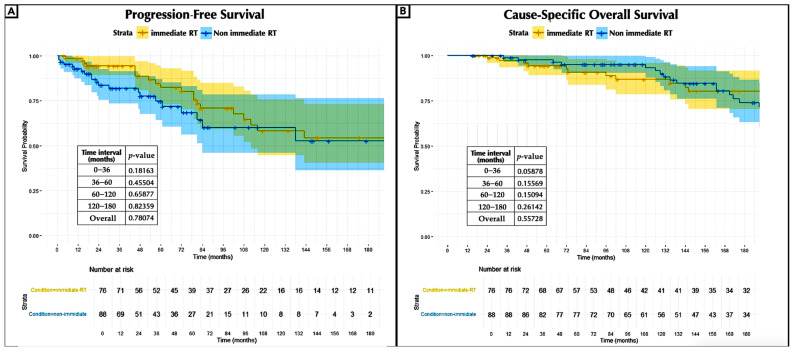
Kaplan–Meier curves of progression-free survival (**A**) and cause-specific overall survival (**B**) comparing adjuvant (immediate RT) and delayed radiotherapy (non-immediate RT) with the number at risk and censored. *p*-value of <0.05 was considered statistically significant; RT, radiation therapy.

In terms of PFS and OS, the survival analysis assessing the timing effect of irradiation demonstrated no significant benefit for radiotherapy administered immediately after surgery compared to non-immediate irradiation (Figure 3A,B).

### 3.5. Illustrative Case

**Figure 4:**
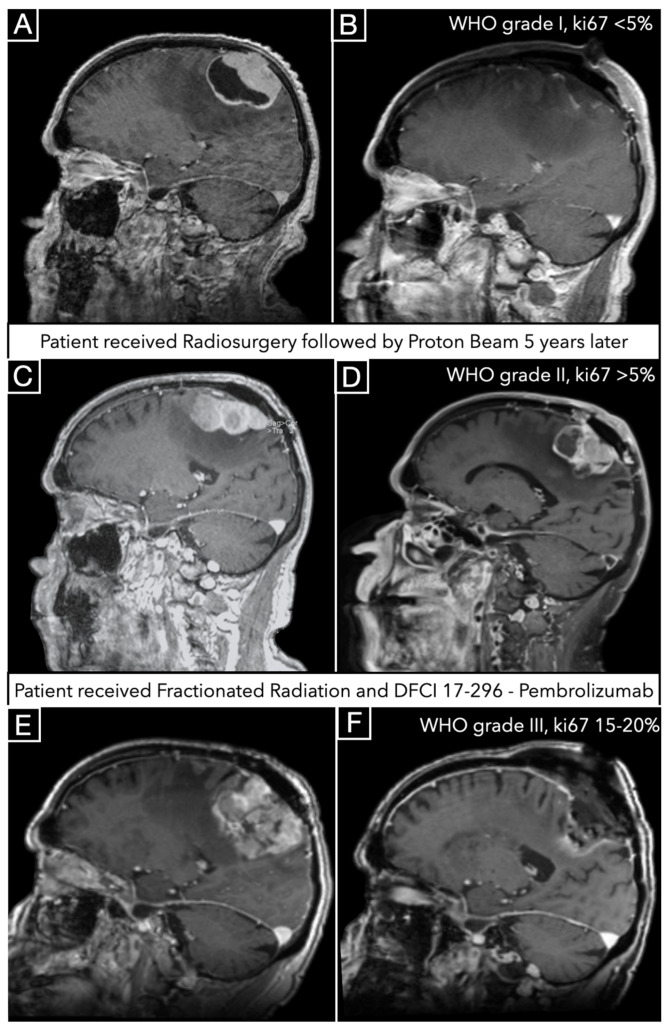
Preoperative and postoperative contrast-enhanced T1-weighted sagittal MR images following the first (**A**,**B**), second (**C**,**D**), and third (**E**,**F**) surgeries.

A 76-year-old male presented with left hemiparesis, and MRI demonstrated a large parasagittal meningioma (Figure 4A). He underwent surgical resection; postoperative MRI is depicted in Figure 3B. The neuropathologist report stated WHO grade I transitional meningioma with ki67 < 5%. Three years later, the patient received proton beam (total dose 12 Gy) for recurrence, and five years later, he received proton beam (total dose 12 Gy) therapy for continuous growth.

The tumor was then resected four years after the proton beam therapy (Figure 4C preoperative, 3D postoperative), and the neuropathologist report indicated WHO grade II atypical meningioma with ki67 higher than 5%. After this second surgery, the patient received additional stereotactic radiation therapy (total dose 50 Gy) and the protocol DFCI 17-296—Pembrolizumab.

Only after one year, the patient returned with remarkable recurrence (Figure 4E), and underwent resection (Figure 4F) with the neuropathologist report of anaplastic WHO grade III meningioma with ki67 15–20%.

Unfortunately, genetic studies were available only after the second operation, which was preceded by both radiosurgery and proton therapy after seven and four years, respectively. The study showed multiple operations. This single study showed multiple genetic aberrations, including RNF43, ATM, and AXL.

### 3.6. Bonferroni-Adjusted Cox-PH Regression Model

**Figure 5:**
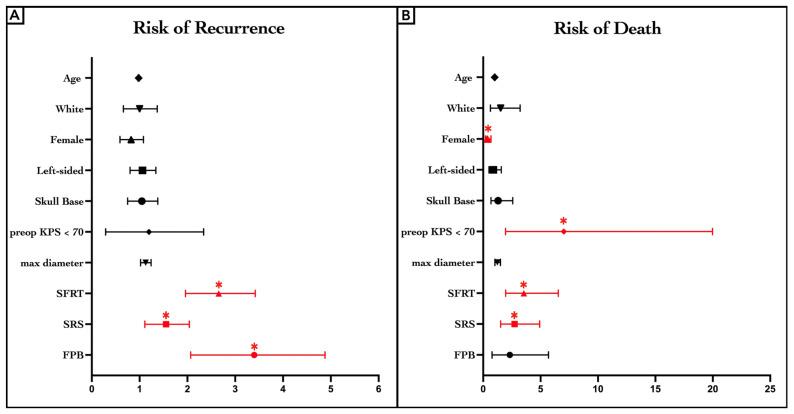
Forest plot of risk of recurrence (**A**) and death (**B**). *: A *p*-value of <0.005 was considered statistically significant; KPS, Karnofsky performance scale.

The Bonferroni-adjusted Cox-PH regression model identified radiation therapies as the only adverse predictors of recurrence risk. The three different radiation modalities were similar. SFRT (hazard ratio [Hz]: 2.598, confidence interval [CI]: 1.963–3.427, *p* < 0.0001 **), SRS (Hz: 1.516, [CI]: 1.110–2.049, *p* = 0.0077 **), and FPB (Hz: 3.241, [CI]: 2.074–4.884, *p* < 0.0001 **) were associated with an increased risk of recurrence compared to the non-radiation group (Figure 5A, Table S2). The results of the Bonferroni-adjusted Cox-PH regression model indicated a higher risk of mortality associated with male sex (Hz: 0.363, [CI]: 0.194–0.682, *p* = 0.0015 *) and preoperative KPS < 70 (Hz: 7.028, [CI]: 1.954–19.98, *p* = 0.0007 **). Furthermore, patients undergoing SFRT (Hz: 3.546, [CI]: 1.960–6.542; *p* < 0.0001 **) and SRS (Hz: 2.748, [CI]: 1.522–4.922; *p* = 0.0007 **) exhibited an increased risk of death compared to those receiving no radiation (Figure 5B, Table S3).

### 3.7. Clinical Endpoints and Complications

Clinical control was achieved in 87% of cases after surgery alone and in 37% of cases after radiation therapies. Pathological malignant progression was identified in only two patients (<1%) post-surgery alone compared to 49 patients (28%) after STR + RT. Newly documented malignant mutations were observed in 5 patients (2%) post-surgery alone and 19 (11%) after STR + RT. After STR and STR + RT, respectively, the KPS at the last follow-up dropped below 70 in 23 patients (9%) and 59 (34%) of patients. Clinical endpoints and surgical and radiation-related complications are listed in Table 2.

## 4. Discussion

### 4.1. Meningioma and Radiotherapy

As early as 1925, Bailey reviewed irradiated brain tumors and concluded that “The outcome of this case is typical of what one may expect in any meningioma, and there is no reason to subject such tumors to roentgen therapy” [9]. In 1938, Cushing and Eisenhardt documented cases of radiation in the treatment of meningioma with limited therapeutic benefit [10]. In 1946, McWhirter favored the use of radiation in a group of meningiomas he labeled as “radiosensitive” [11], only to find later on, reviewing his pathology, that some of the tumors considered sensitive were not meningiomas. He later revised his conclusions, concluding that the “typical slow-growing meningiomas are radio-insensitive” [12]. In a later, more advanced and extensive study of radiotherapy in managing meningiomas, King and colleagues concluded that they “failed to demonstrate convincingly that radiotherapy has a beneficial effect in reducing the rate of recurrence” [13]. As late as 2003, Mathiesen and co-workers reported that 75% of all subtotally resected and radiated meningiomas recurred during a mean follow-up of 9 years [14]. Hence, the quest was to achieve total removal to minimize recurrence as it was directly related to the extent of surgical resection [2]. Meningioma treatment for decades was exemplified by Bucy’s statement, “Meningioma are benign tumors; if they are completely removed, they do not recur” [15].

Advanced radiation methods advanced the practice of irradiating meningiomas, and this approach became commonly and widely used for postoperative residuals or even as a primary treatment, supporting its safety profile and potential efficacy, thus attracting increased interest in achieving total tumor resection [16,17]. In a prospective phase II trial (NRG/RTOG 0539), Rogers et al. raised a question about using radiation as an adjuvant after subtotal resection for benign meningioma [8]. Our study compared the extended long-term outcomes of WHO grade I meningiomas treated with surgery alone with those treated with surgery combined with radiotherapy over three decades.

### 4.2. Progression-Free Survival and Clinical Control

The current literature documents an advantage of using irradiation via various modalities in achieving clinical control and reducing recurrence rates, particularly for residual tumors [18,19,20,21,22].

One representative article is a multicenter study of 675 patients with posterior fossa meningiomas treated with surgery and radiation, in which clinical control was achieved in 91.2% of cases at a mean follow-up of 60.1 months, with an actuarial PFS rate of 95%, 92%, and 81% at 3, 5, and 10 years, respectively, but with a continuing declining rate [23]. Some authors reported frequent tumor control with adjuvant radiation after meningioma resection [22].

Most of the studies asserting advantages of the use of radiation for postoperative residuals have relatively short follow-up periods of around 5 years.

Our findings differ from these reports, as they are based on a more extended follow-up period with a mean follow-up of 127.28 months and a median of 103.5 months. After irradiation, clinical control was achieved in 37%, with a high recurrence rate (88%). The actuarial PFS rates at 3, 5, 10, and 15 years were 59%, 43%, 23%, and 16%, compared to 91%, 85%, 77%, and 70% after surgery alone. A notable difference was detected as early as 3 years of follow-up. Our rates of clinical control and PFS after irradiation are substantially lower than in the STR group and even lower than those reported in the literature regarding the natural history of non-radiated residuals [3,8,24,25].

### 4.3. Survival Rates

Rowe and colleagues reported an actuarial survival rate of 53% at 15 years in an extensive series of tumors treated with gamma-knife radiosurgery, with meningioma progression as the principal cause of death in 69% of patients [26]. Other investigators reported a similar mortality rate of 137 out of 249 (47%) at a median of 8.7 years [27]. Because death occurs for a variety of reasons other than a meningioma, a cause-specific mortality rate would be more accurate and conclusive. In a relatively short median follow-up of 4 years, an analysis of 424 WHO grade I meningiomas out of 1045 treated with adjuvant radiosurgery reported actual disease-specific survival rates of 98.9%, 96.2%, and 96.2% at 5, 10, and 15 years, respectively [28]. This finding is comparable with other reports that have a short follow-up period [28,29,30].

The extended long-term survival rate in our study shows a divergence from these earlier, more favorable results. The cause-specific OS rates at 3, 5, 10, 15, and 20 years were 98%, 97%, 93%, 85%, and 76% after radiation, with mortality at 5, 10, and 15 years of 21%, 18%, 16%, and 45% and an overall mortality rate of 26%. In contrast, in the surgery-alone cohort, cause-specific OS rates at 5, 10, 15, and 20 years were 98%, 97.6%, 97.6%, 97.6%, and 96%, consistent with the reported natural history of benign meningiomas [8,25,31]. These findings do not demonstrate a survival benefit associated with radiotherapy compared to the natural history of benign residual tumors after subtotal resection.

Furthermore, the Bonferroni-adjusted Cox-PH regression model revealed that all the different irradiation modalities are related to recurrence, while SFRT and SRS are associated with an increased risk of mortality in multivariable analysis.

### 4.4. Early vs. Delayed Adjuvant Radiotherapy

Our survival analysis assessing the effect of irradiation timing showed no significant benefit of immediate postoperative radiotherapy compared to delay in terms of PFS and OS. In this regard, deferring radiotherapy until tumor progression is documented and may represent a more judicious approach. This stance contrasts with that of other authors who advocate for early radiotherapy in the management of residual tumors shortly after surgery [32,33]. These findings may be influenced by the inclusion of residual tumors that might not have naturally progressed.

### 4.5. Malignant Progression

The literature on radiated meningiomas has dismissed the concern of malignant transformation after irradiation, considering its low incidence negligible [18,34,35]. Some accounts have reported a 0% incidence [27,36]. However, previous research has suggested a potential association between irradiation and malignant transformation in benign tumors. The ascribed mechanism focused on the damage by radiation to DNA, interference with the DNA repair mechanism, and genetic instability. New and accumulated mutations result in malignant progression, along with other mechanisms, such as enriching the tumor stem cells, epigenetic changes, alterations in the cellular microenvironment, and clonal selection [37,38].

An early clinical observation demonstrated a pattern of aggressive growth of meningiomas at the time of failure after radiosurgery [39]. In the present cohort, the observed rate of malignant progression in the irradiated cohort was notably elevated, particularly considering that the rate of malignant progression in non-irradiated tumors is only 1%.

### 4.6. Study Limitations

This study has the intrinsic limitations of a retrospective design in one institution, including a vulnerability to selection and recall biases. Additionally, several factors might affect the results: the lack of uniform criteria for radiation and information to assess the extent of resection via the Simpson grading system, the evolving radiation therapy techniques over the extended study period, and the involvement of multiple surgeons with different levels of expertise. Furthermore, in recent years, there have been pivotal advances in genetic and molecular studies, which have had a major influence on the classification, management, and outcomes of meningiomas and subsequently the means of treating them [40,41].

## 5. Conclusions

Comparing the extended long-term outcomes of non-irradiated residual WHO grade I meningiomas with irradiated ones, the irradiated group has a lower control and PFS and a higher cause-specific mortality and malignant transformation. These results suggest a reevaluation of current treatment guidelines, emphasizing a more conservative approach to RT in low-risk WHO grade I meningiomas.

## Figures and Tables

**Table 1:** Data of 432 patients undergoing subtotal resection of WHO grade I meningioma (N = 436).

Data	Value
Timeframe	1990 through 2023
Number of patients	432
Number of meningiomas	436
In female patients, *n.* (%)	319 (73%)
In male patients, *n.* (%)	117 (27%)
Mean age (years ± SD)	69 ± 13.9
Race, *n.* (%)	
Caucasian	363 (83%)
Black	21 (5%)
Asian	7 (2%)
Hispanic	21 (5%)
Not available	24 (5%)
Side, *n.* (%)	
Left	198 (45%)
Right	185 (43%)
Median/bilateral	53 (12%)
Location, *n.* (%)	
Vault	146 (33%)
Skull Base	
Anterior	150 (34%)
Middle	81 (19%)
Posterior	57 (13%)
Intraventricular	2 (1%)
Average maximum diameter (cm ± SD)	3.4 ± 1.4
Preoperative Karnofsky Performance Scale score	
≤70	40 (9%)
>70	396 (91%)
Pretreatment neurological deficits	296 (68%)
Pretreatment hydrocephalus	6 (1%)
Intraoperative complications, *n.* (%)	
Vascular injury/hemorrhage/ischemia	9 (2%)
Irradiated, *n.* (%)	176 (40%)
MIB-11/Ki-67, *n.* (%)	
≤5%	163 (37%)
>5%	64 (15%)
Not available	209 (48%)
Genetic profile, *n.* (%)	
Benign/monosomy 22	78 (18%)
Malignant multiple mutations	25 (6%)
Not available	333 (76%)

n., number; SD, standard deviation.

**Table 2:** Extended long-term outcomes, clinical endpoints, and complications of 436 patients with postoperative residual WHO grade I meningioma, divided in 2 groups: surgery alone (STR) and surgery plus radiation therapy (STR + RT).

Data	STR (n. 260)	STR + RT (n. 176)
Actual median PFS (months/years)	59/4.9	47.5/3.9
Actuarial PFS (%)		
3 years	91%	59%
5 years	85%	43%
10 years	77%	23%
15 years	70%	16%
Actual median cause-specific OS (months/years)	112/9.3	150/12.5
Actuarial cause-specific OS (%)		
3 years	98%	98%
5 years	97.6%	97%
10 years	97.6%	93%
15 years	97.6%	85%
20 years	96%	76%
Mortality rate (%)		
Overall	4%	26%
36 months	10%	21%
36–60 months	6%	18%
60–120 months	0%	16%
120–180 months	0%	45%
Clinical control *n*. (%)	225 (87%)	68 (37%)
Malignant progression *n*. (%)	2 (1%)	49 (28%)
to WHO II	2 (1%)	39 (22%)
to WHO III	/	3 (2%)
to WHO II–III	/	7 (4%)
New malignant mutations *n*. (%)	5 (2%)	19 (11%)
KPS at the last follow-up		
≤70	23 (9%)	59 (34%)
>70	237 (21%)	117 (66%)
Postoperative complications *n.* (%)		
Vascular	20 (8%)	4 (2%)
Cranial nerve palsy	26 (10%)	18 (10%)
Abscess/wound infection	2 (1%)	3 (2%)
Hydrocephalus/CSF leak	10 (4%)	2 (1%)
Motor impairment	4 (2%)	3 (2%)
Seizure	7 (3%)	2 (1%)
RT complications n. (%)		
Radiation necrosis	/	67 (38%)
T2 signal hyperintensity	/	118 (67%)
Pituitary dysfunction	/	29 (17%)
Visual impairment	/	53 (30%)
Hydrocephalus	/	22 (13%)

cm, centimeter; CSF, cerebrospinal fluid; KPS, Karnofsky performance status; n., number; OS, overall survival; PFS, progression-free survival; RT, radiation treatment; SD, standard deviation; STR, subtotal resection.

## Data Availability

Access to the raw dataset can be requested by contacting the corresponding author.

## Supplementary Materials

The following supporting information can be downloaded at https://www.mdpi.com/article/10.3390/jcm14165829/s1, Table S1: Recurrence rates, Table S2: Bonferroni-adjusted Cox proportional-hazards regression model for risk of recurrence, Table S3: Bonferroni-adjusted Cox proportional-hazards regression model for risk of death.

## Abbreviations

The following abbreviations are used in this manuscript:

CI	Confidence Interval
FPB	Fractionated Proton Beam Therapy
Hz	Hazard Ratio
MRI	Magnetic Resonance Imaging
OS	Overall Survival
PFS	Progression-Free Survival
RBE	Relative Biological Effectiveness
RT	Radiation Therapy
SFRT	Fractionated Stereotactic Conformal Radiotherapy
SRS	Stereotactic Radiosurgery
STR	Subtotal Resection
