# Supplementary material for: Impact of Irradiation on Post-Surgical Residuals of WHO Grade I Meningioma
Source: J Clin Med. 2025 Aug 18;14(16):5829. doi: 10.3390/jcm14165829 (PMC12386829; doi:10.3390/jcm14165829)
Supplement: Supplementary file 1 [file jcm-14-05829-s001.zip › jcm-3786352-supplementary.pdf]

Table S1. Recurrence Rates

Data	STR (n. 260)	STR+RT (n. 176)
Recurrence, n. (%)		
1st	86 (33%)	155 (88%)
2nd	7 (3%)	72 (41%)
3rd	1 (1%)	40 (23%)
4th	/	15 (9%)
5th	/	4 (2%)
6th	/	2 (1%)
7th	/	2 (1%)
Surgeries for recurrence, n.		
2	35 (13%)	87 (49%)
3	3 (1%)	51 (29%)
4	/	18 (10%)
5	/	6 (3%)
6	/	2 (1%)
7	/	2 (1%)
8	/	1 (1%)
Mean time between recurrences (months / years)		
1st recurrence	89.8 / 7.4	62.1 / 5.1
2nd recurrence	50.5 / 4.1	44.9 / 3.7
3rd recurrence	52 / 4.3	14 / 1.1
4th recurrence	/	11.2 / <1
5th recurrence	/	5.7
6th recurrence	/	5.5
7th recurrence	/	2
Mean rate of tumor growth (cm)		
1st recurrence	2	2.5
2nd recurrence	2.8	2.9
3rd recurrence	4	3.3
4th recurrence	/	3.4
5th recurrence	/	3.9
6th recurrence	/	4.7

cm: centimeter, n: number; RT: Radiation; STR: Subtotal Resection.

Table S2. Bonferroni-adjusted Cox Proportional-Hazards Regression Model for Risk of Recurrence

**Variable**	**Hazard Ratio**	**Lower CI**	**Upper CI**	**p-Value**
Age	0.988	0.978	0.999	0.0325
Race (White)	0.971	0.669	1.374	0.8742
Female	0.801	0.599	1.081	0.1396
Left-sided	1.042	0.804	1.348	0.7562
Skull Base	1.014	0.750	1.382	0.9283
Preop KPS <70	0.968	0.293	2.349	0.9499
Max diameter	1.128	1.025	1.240	0.0130
SFRT	2.598	1.963	3.427	**<0.0001****
SRS	1.516	1.110	2.049	**0.0077****
FPB	3.241	2.074	4.884	**<0.0001****

CI: confidence interval; FPB: fractionated proton beam therapy; Preop KPS: Preoperative Karnofsky Performance Status; SFRT: fractionated stereotactic conformal radiotherapy; SRS stereotactic radiosurgery. *: A p-value of <0.005 set as statistically significant

Table S3. Bonferroni-adjusted Cox Proportional-Hazards Regression Model for Risk of Death

**Variable**	**Hazard Ratio**	**Lower CI**	**Upper CI**	**p-Value**
Age	1.009	0.984	1.035	0.4699
Race (White)	1.522	0.639	3.227	0.3034
Female	0.363	0.194	0.682	**0.0015***
Left-sided	0.895	0.500	1.589	0.7071
Skull Base	1.308	0.680	2.587	0.4285
Preop KPS <70	7.028	1.954	19.98	**0.0007****
Max diameter	1.249	1.028	1.506	0.0218
SFRT	3.546	1.960	6.542	**<0.0001****
SRS	2.748	1.522	4.922	**0.0007****
FPB	2.327	0.771	5.694	0.0910

CI: confidence interval; FPB: fractionated proton beam therapy; Preop KPS: Preoperative Karnofsky Performance Status; SFRT: fractionated stereotactic conformal radiotherapy; SRS stereotactic radiosurgery. *: A p-value of <0.005 set as statistically significant
